# A case of toxic epidermal necrolysis associated with vonoprazan, a potassium-competitive acid blocker

**DOI:** 10.1016/j.jdcr.2025.12.028

**Published:** 2025-12-26

**Authors:** Donna Pham, Kathleen L. Miao, Yasmin Gutierrez, Scott Worswick

**Affiliations:** a University of California, Riverside School of Medicine, Riverside, California; b Keck School of Medicine of University of Southern California, Los Angeles, California; c Department of Dermatology, Keck School of Medicine, University of Southern California, Los Angeles, California

**Keywords:** adverse drug reaction, gastroesophageal reflux disease, potassium-competitive acid blocker, severe cutaneous adverse reaction, Stevens-Johnson syndrome, streptococcus pyogenes, toxic epidermal necrolysis, vonoprazan

## Introduction

Vonoprazan, a novel potassium-competitive acid blocker (P-CAB), is approved by the United States (U.S.) Food and Drug Administration for the treatment of various acid-related gastrointestinal disorders.^1^ First introduced in Japan in 2015, vonoprazan received U.S. approval in 2022 as part of a *Helicobacter pylori* (*H pylori*) eradication regimen, later expanding to erosive esophagitis and gastroesophageal reflux disease.^2^

Stevens-Johnson syndrome (SJS) and toxic epidermal necrolysis (TEN) constitute a spectrum of severe mucocutaneous reactions marked by extensive keratinocyte necrosis, epidermal detachment, and high morbidity and mortality.^3^ We present a case of TEN associated with vonoprazan use.

## Case report

A 26-year-old man with a history of gastroesophageal reflux disease presented to an outside hospital with a progressive blistering eruption. Four days prior to admission, he developed fever, chills, and sore throat, followed by a rash 2 days later. On the day of admission, flaccid blisters developed over the rash, accompanied by mucosal involvement. His symptoms began approximately 1-2 weeks after initiating a four-dose trial of 20 mg vonoprazan for gastroesophageal reflux disease.

On admission, the patient was febrile (104 °F), tachycardic, and normotensive. Skin biopsy revealed full-thickness epidermal necrosis consistent with TEN. A broad infectious workup, including a chest radiograph, respiratory viral panel, and throat cultures for Group A, C, and *G streptococci*, was negative.

**Fig 1:**
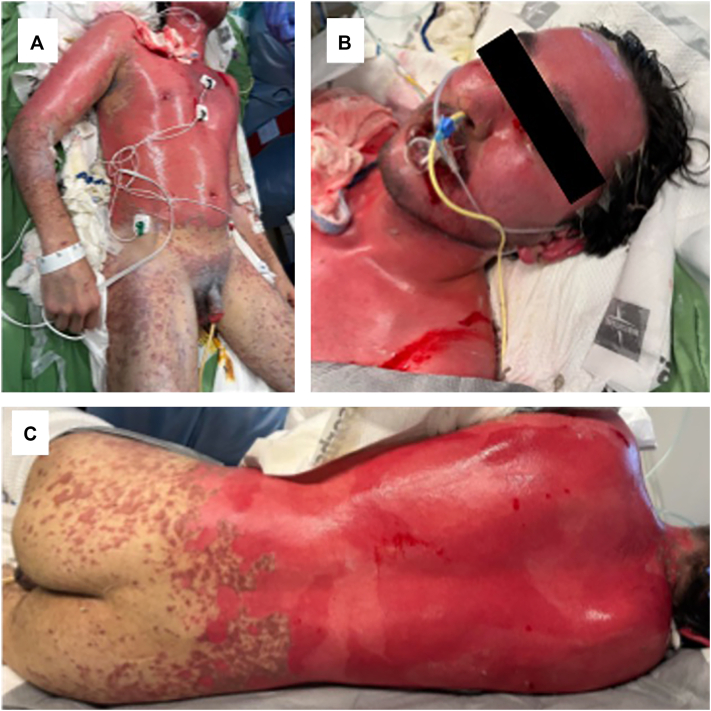
**A-C,** Clinical photographs of the patient upon admission (hospital day 6) to our burn unit, demonstrating extensive skin sloughing with involvement of the eyes, mouth, genital, and perianal regions, overall affecting about 54% BSA involvement. In the surrounding areas, there was diffuse involvement of dusky violaceous macules and coalescing patches, with flaccid bullae with positive Nikolsky sign. *BSA*, Body surface area.

The patient was treated with intravenous immunoglobulin (80 g/d) for 3 days, high-dose corticosteroids (methylprednisolone 1000 mg/d for 3 days, followed by 125 mg/d for 2 days), and 2 subcutaneous doses of etanercept (50 mg each). On hospital day 6, the patient was transferred to our institution’s burn unit for further management. Upon admission, 54% of body surface area was involved, affecting the trunk and bilateral upper extremities with extensive mucocutaneous involvement (Fig 1). Mucosal sloughing and desquamation of the lips were present, with all 4 mucosal surfaces involved (ocular, oral, penile, and perianal mucosa). Given the patient already received multiple systemic immunomodulatory therapies, further systemic treatment was deferred. Silver oxysalt dressings were applied to the affected areas, and supportive care included fluid resuscitation and pain medications. Ophthalmology confirmed ocular involvement and prescribed ofloxacin, cyclosporine, and prednisolone eyedrops with lubricating ointment. During hospitalization, the patient developed transaminitis; however, this normalized spontaneously and was deemed secondary to TEN-associated systemic involvement.^4^ By hospital day 10, the patient’s skin had significantly re-epithelialized with no signs of further progression.

Vonoprazan was identified as the likely causative agent, as it was the only newly initiated medication following a comprehensive review of the patient’s medications, supplements, and any over-the-counter products. However, given the initial presentation of a high fever and sore throat, a comprehensive infectious workup ─ including antistreptolysin-O (ASO) ─ was pursued to rule out other causes.

**Fig 2:**
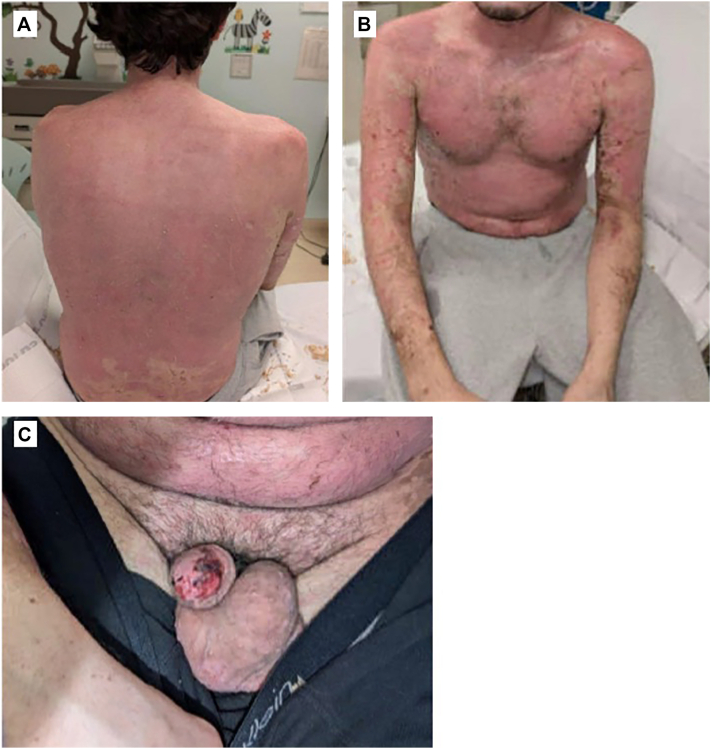
**A-C,** Clinical photographs of the patient 4 days after discharge with significant clinical improvement, with approximately 0.5% BSA remaining open. *BSA*, Body surface area.

Laboratory results revealed an elevated ASO titer. Given the patient’s preceding symptoms of fever, sore throat, cough, and congestion, it was unclear whether these represented a prodrome to SJS/TEN or were due to *Group A streptococcal* (GAS) pharyngitis. As a precaution, the patient was empirically treated with amoxicillin for GAS pharyngitis on day 11. He began tolerating a regular diet and was discharged on day 12. At follow up 4 days later, he demonstrated significant clinical improvement, with approximately 0.5% body surface area involvement remaining (Fig 2).

## Discussion

This case represents a suspected instance of vonoprazan-induced TEN, involving a medication not previously implicated in TEN when used as monotherapy. The temporal relationship between vonoprazan initiation and symptom onset, in the absence of other new medications, supports a likely drug-induced etiology. Although elevated ASO titers and preceding upper respiratory symptoms raised the possibility of a streptococcal trigger for reactive infectious mucocutaneous eruption, this is considered less likely given the rarity of streptococcal-induced reactive infectious mucocutaneous eruption, the predominance of cutaneous symptoms, and the patient’s negative throat culture earlier in the clinical course.

P-CABs include fexuprazan, keverprazan, revaprazan, tegoprazan, and vonoprazan, with linaprazan and zastaprazan under investigation.^2^ Among these, vonoprazan is the most extensively studied and the only P-CAB approved in the U.S. Despite its growing clinical use, limited literature describes cutaneous adverse effects associated with P-CABs. One study reported a single case of SJS/TEN associated with vonoprazan/amoxicillin/clarithromycin (VONOSAP).^5^ Otherwise, reported cutaneous adverse events with vonaprazan monotherapy have been limited to erythema multiforme (*n* = 6) and maculopapular eruptions (*n* = 11). SJS/TEN, erythema multiforme, and maculopapular drug eruptions share overlapping pathophysiology as delayed-type hypersensitivity reactions. Both SJS/TEN and erythema multiforme involve CD8+ cytotoxic T-cell mediated keratinocyte injury. However, SJS/TEN is distinguished by extensive keratinocyte apoptosis driven by granulysin, Fast ligand, perforin, and granzyme B, resulting in significantly higher morbidity.^3^ While SJS/TEN has been reported as a rare adverse event associated with proton pump inhibitors, there are currently no published cases implicating P-CAB monotherapy in SJS/TEN.

Although infections, particularly *Mycoplasma pneumoniae*, are known SJS/TEN precipitants, *Streptococcus pyogenes* is an exceedingly rare trigger. To date, only 1 published case has attributed TEN with confirmed GAS pharyngitis.^6^ Another case reported an incidental positive rapid strep test in a patient with a similar prodrome of sore throat, but did not identify GAS as the likely causative agent.^7^ More often, GAS is associated with reactive infectious mucocutaneous eruption, a condition that can mimic TEN clinically but typically has predominant mucosal involvement and usually presents in pediatric patients.^8^, ^9^, ^10^ Although the association between GAS infection and TEN remains poorly defined, these rare reports suggest it may represent a potential, albeit extremely uncommon, trigger.

In conclusion, we present what is, to our knowledge, the first documented case of TEN associated with vonoprazan monotherapy. The temporal relationship, clinical presentation, and lack of alternative etiologic agents support vonoprazan as the likely trigger. In the presence of clinical symptoms with a positive ASO titer, GAS may also be considered as a causative agent, another exceedingly rare trigger. As the clinical use of P-CABs continues to expand, it is essential that clinicians remain vigilant for severe cutaneous adverse reactions. Further investigation into the potential for SJS/TEN within this drug class is warranted to ensure patient safety and guide future prescribing practices.

## Conflicts of interest

None disclosed.
